# Pharmacological Activity and Clinical Use of PDRN

**DOI:** 10.3389/fphar.2017.00224

**Published:** 2017-04-26

**Authors:** Francesco Squadrito, Alessandra Bitto, Natasha Irrera, Gabriele Pizzino, Giovanni Pallio, Letteria Minutoli, Domenica Altavilla

**Affiliations:** ^1^Section of Pharmacology, Department of Clinical and Experimental Medicine, University of MessinaMessina, Italy; ^2^Department of Biomedical and Dental Sciences and Morphofunctional Imaging, University of MessinaMessina, Italy

**Keywords:** adenosine A2 receptors, DNA, oligonucleotides, PDRN, wound healing

## Abstract

PDRN is a proprietary and registered drug that possesses several activities: tissue repairing, anti-ischemic, and anti-inflammatory. These therapeutic properties suggest its use in regenerative medicine and in diabetic foot ulcers. PDRN holds a mixture of deoxyribonucleotides with molecular weights ranging between 50 and 1,500 KDa, it is derived from a controlled purification and sterilization process of *Oncorhynchus mykiss* (Salmon Trout) or *Oncorhynchus keta* (Chum Salmon) sperm DNA. The procedure guarantees the absence of active protein and peptides that may cause immune reactions. *In vitro* and *in vivo* experiments have suggested that PDRN most relevant mechanism of action is the engagement of adenosine A_2A_ receptors. Besides engaging the A_2A_ receptor, PDRN offers nucleosides and nucleotides for the so called “salvage pathway.” The binding to adenosine A_2A_ receptors is a unique property of PDRN and seems to be linked to DNA origin, molecular weight and manufacturing process. In this context, PDRN represents a new advancement in the pharmacotherapy. In fact adenosine and dipyridamole are non-selective activators of adenosine receptors and they may cause unwanted side effects; while regadenoson, the only other A_2A_ receptor agonist available, has been approved by the FDA as a pharmacological stress agent in myocardial perfusion imaging. Finally, defibrotide, another drug composed by a mixture of oligonucleotides, has different molecular weight, a DNA of different origin and does not share the same wound healing stimulating effects of PDRN. The present review analyses the more relevant experimental and clinical evidences carried out to characterize PDRN therapeutic effects.

## Introduction

In the last decade great attention has been dedicated by pharmacologists to the characterization of the pharmacological properties of substances that are needed and produced by living organisms. DNA derived drugs might have relevant therapeutic effects in a clinical setting. In this context, there are two drugs named PDRN (polydeoxyribonucleotide) and defibrotide that share the same “natural” source: DNA (Altavilla et al., 2009; Keating, 2014). However, they differ in DNA origin, molecular weight, and manufacturing procedures; as a consequence, they have different pharmacological properties, mechanism(s) of action and, eventually, clinical effects. While some excellent reviews on defibrotide have been recently published (Pescador et al., 2013; Richardson et al., 2013), there is only one dedicated to PDRN that dates back to 2009 (Altavilla et al., 2009). However, the last years have testified an increased interest in PDRN with a large number of experimental and clinical studies carried out with this DNA derived drug. Therefore, aim of this paper is to review and up-date the experimental and clinical work carried out on PDRN.

### PDRN chemistry

PDRN is a proprietary and registered DNA derived drug. It is a mixture of deoxyribonucleotides with molecular weights between 50 and 1,500 KDa and it is derived from *Oncorhynchus mykiss* (Salmon trout) or *Oncorhynchus keta* (Chum Salmon) sperm DNA. The most represented molecular weight is 80–200 KDa, with a peak of the Gaussian distribution at 132 KDa. PDRN has a higher molecular weight compared to defibrotide (16.5 ± 2.5 KDa) (Guglielmelli et al., 2012; Richardson et al., 2013). Furthermore, defibrotide is derived from the porcine intestinal mucosal DNA (Table 1). PDRN is extracted and purified at high temperature, a procedure that allows to recover a >95% pure active substance with inactivated proteins and peptides. This latter guarantees the safety of the product and the absolute lack of any immunological side effect. Indeed, the source of raw material (cells vs. organs) is of particular importance: spermatozoa are the most appropriate cells to provide highly purified DNA without risk of impurity such as peptides, proteins and lipids which can remain from the somatic cells.

**Table 1:** **The most important characteristics of PDRN and defibrotide**.

**Composition**	**50% Double stranded deoxyribonucleotides**	**90% Single stranded oligonucleotides**
DNA source	DNA sperm from *Oncorhynchus mykiss* (Salmon Trout) or *Oncorhynchus keta* (Chum Salmon)	Porcine intestinal mucosa DNA
Molecular weight	50–1,500 KDA	16–20 KDA
Main activities	Tissue repairing, wound healing, anti-ischemic, and anti-inflammatory effects	Profibrinolytic, antithrombotic and thrombolytic effects, anti-ischemic, and anti-rejection effects, anti-angiogenetic
Mode of action	Activation of adenosine A_2A_ receptors	Reduction of endothelial activation

### PDRN pharmacokinetics

The pharmacokinetics of PDRN has been evaluated after a single intraperitoneal administration of 8 mg/kg in rat. Measurable levels of PDRN were observed 15 min post-injection, and peak levels 1 h after drug administration, with a bioavailability of 90%. Drug levels then decreased progressively, being PDRN still measurable (0.137 μg/ml) 6 h following injection. The half-life is of 3 h and it is not influenced by dosage. As analyzed below, the drug stimulates the initiation of a cascade of events involving a number of transduction effectors that last much more than its plasma half–life. Therefore, the pharmacodynamics effects of PDRN may be much longer than anticipated by its pharmacokinetic profile. Due to its chemical structure, plasmatic carrier proteins do not bind PDRN, but it is found free in plasma. The distribution of the free drug depends upon tissue blood flow, being higher in those organs with elevated blood supply. The drug is not metabolized by the liver and there is no evidence for a first-passage metabolism. Instead, the drug is mainly degraded by unspecific plasma DNA nucleases, or by nucleases bound to cell membranes leading to oligo and mononucleotides. From a pharmacodynamics point of view, this event is of paramount importance: in fact, PDRN degradation gives rise to the formation of nucleosides and nucleotides that become available for the main activity: the binding to the adenosine A_2A_ receptor. PDRN fragments are then excreted in the urine (~65%) and to a lesser extent in the feces.

The pharmacokinetics of PDRN has been also studied in healthy volunteers after intramuscular administration (5.625 mg). The results of this study showed a pharmacokinetic profile overlapping to that observed in experimental animals: more specifically peak levels at ~1 h; a half-life of ~3.5 h, with a bioavailability in the range of 80/90%.

### Pharmacological properties of PDRN: experimental *in vitro* studies supporting the mode of action

**Figure 1:**
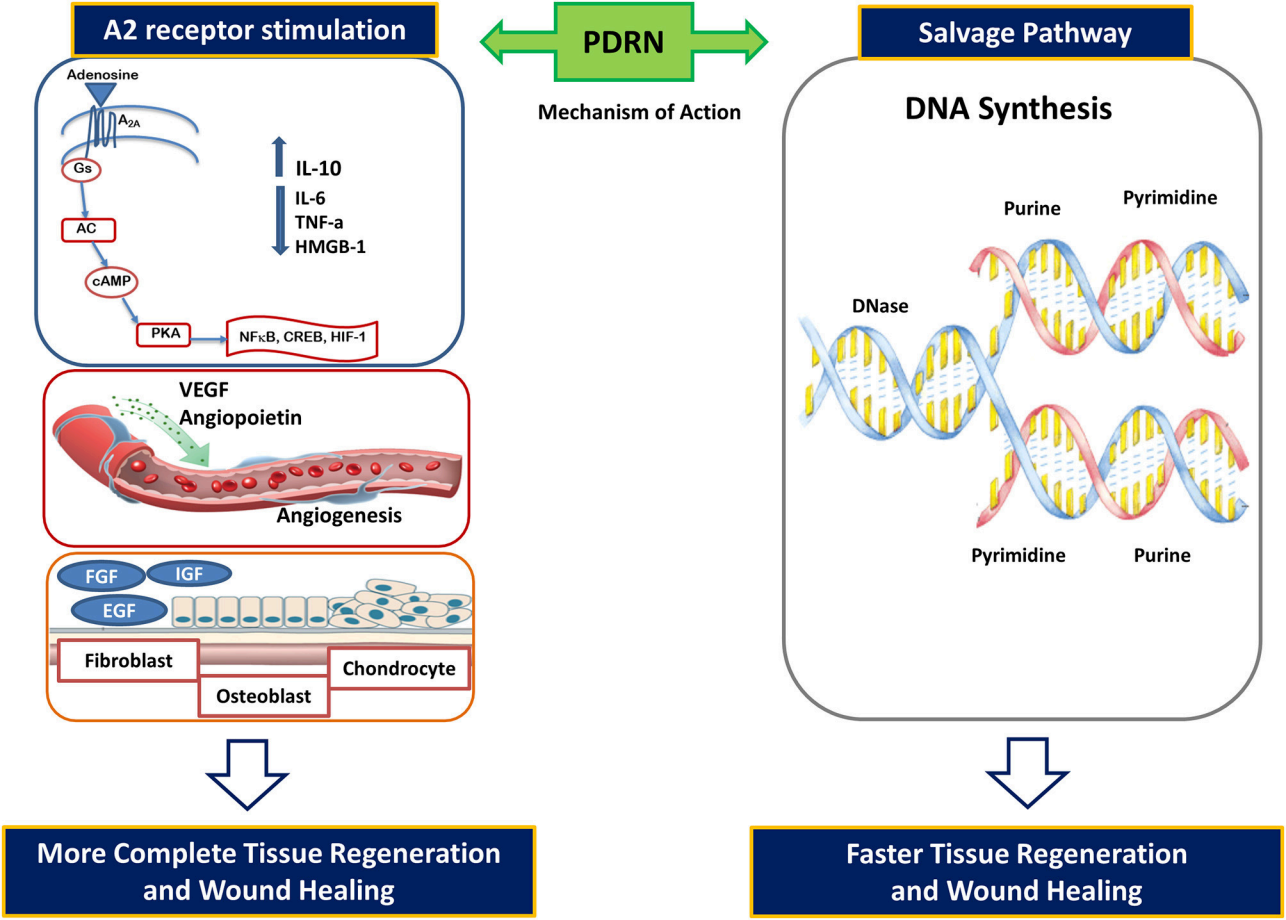
**PDRN mode of action: activation of adenosine A2 receptors and the “salvage pathway”**.

Adenosine activates four distinct adenosine receptors indicated as A_1_, A_2A_, A_2B_, and A_3_. These receptors are widely expressed and implicated in several physiological and pathological functions. The A_2A_ receptor play a central role in modulating inflammation, oxygen consumption, ischemia, cell growth, and angiogenesis. PDRN was compared to adenosine in primary cultures of human skin fibroblasts (Thellung et al., 1999): both PDRN and adenosine induced cell growth. The effects of PDRN were abolished by the concomitant incubation with an adenosine A2 receptor antagonist, 3,7-Dimethyl-1-propargylxanthine (DMPX). Indeed, DMPX has greater affinity for A2_A_ than for A2_B_ receptor subtype. This leads to hypothesize that PDRN may preferentially act on the adenosine A2_A_ receptor thus suggesting the involvement of this receptor subtype in PDRN effects. Indeed, it can be speculated that PDRN may represent a pro-drug able to generate active deoxyribonucleotides, nucleosides, and bases exerting their pharmacological effects interacting with the A2_A_ receptor (Figure 1).

In further experiments, cultured fibroblasts were loaded with radioactive amino acids in the presence of PDRN (Sini et al., 1999). Cell growth was accompanied by internalization of PDRN-derived nucleotides to offer purine and pyrimidine rings for the “salvage pathway.” In fact, damaged or hypoxic tissue very often cannot undergo to the DNA “*de novo*” synthesis (Figure 1). Under these conditions, salvage pathways operate to recover bases and nucleosides generated from the breakdown of DNA and RNA. The salvaged bases can be then transformed into nucleotides and reincorporated into DNA. PDRN generates nucleotides and nucleosides that can contribute to DNA formation, thus reactivating normal cell proliferation and growth pattern (Figure 1).

PDRN stimulatory effect on cell growth was also investigated in human cultured osteoblasts (Guizzardi et al., 2003). PDRN (20 mg/ml) promoted cell proliferation with a concomitant increase in alkaline phosphatase and DMPX suppressed this effects. PDRN has been also tested in primary chondrocytes (Gennero et al., 2013), where induced a physiological accumulation of the extracellular matrix with reduced proteoglycan degradation, reducing matrix metalloproteinases 2 and 9. Moreover, PDRN synergizes with glucosamine in reducing extracellular matrix gene expression, thus reducing its degradation (Avantaggiato et al., 2014). These evidences candidates the DNA derived drug as a potential therapy in regenerative cartilage treatment.

Additionally, PDRN may also protect cells from UV-induced DNA damage. Exposure of human dermal fibroblasts to ultraviolet B radiation causes accumulation of dangerous photoproducts such as cyclobutane pyrimidine dimers (CPDS). PDRN addition to the cell culture immediately after irradiation resulted in p53 protein activation and in enhancing DNA repair likely due to the priming of the salvage pathway (Belletti et al., 2007).

Another important aspect useful in regenerative medicine could be related to PDRN ability to increase the proliferation of human pre-adipocytes (Raposio et al., 2008). As a matter of fact, adipose tissue represents a relevant source of adult stem cells, thus PDRN may be used for therapeutic and regenerative purposes.

Collectively these *in vitro* evidences support the concept that PDRN engages the A_2A_ and has cell proliferative and regenerative effects.

### Pharmacological properties of PDRN

#### Tissue repairing, wound healing and therapeutic angiogenesis

It has been suggested that PDRN accelerates the repair and the growth of bone tissue (Guizzardi et al., 2007). Furthermore, PDRN effects were investigated in a model of diabetes-impaired wound healing (Galeano et al., 2008). Disorders in wound healing are very common in diabetes and they represent a major clinical challenge: in fact, there is still an unmet need for a safe treatment able to counteract this clinical situation. PDRN improved the skin repair process and enhanced wound-breaking strength in diabetic animals. This effect was supported by a marked increase in the expression of Vascular Endothelial Growth Factor (VEGF), a master regulator of angiogenesis that is impaired in diabetes-related wound disorders (Galeano et al., 2008). Angiogenesis improvement was confirmed by an increase in CD31, transglutaminase-II, and angiopoietin, factors contributing to new vessel formation. The healing-promoting effect was abrogated by DMPX, thus suggesting the involvement of the adenosine A_2A_ receptor. The positive effect on wound healing and angiogenesis is a characteristic feature of PDRN that is not shared by other DNA-derived drugs that have different DNA origin, molecular weight and manufacturing process. In fact, defibrotide (Keating, 2014) inhibits angiogenesis (Table 1). This observation led us to speculate that the eventual approval of a DNA containing drug biologically equivalent to the registered PDRN (i.e., a PDRN bioequivalent drug) should be considered with extreme caution. To claim the same therapeutic indication, a new formulation should be more tested as a “bioequivalent drug.” More specifically any other DNA containing drug with either smaller or higher molecular weight of DNA polymer should be granted market approval on basis of its own efficacy, safety and clinical data, comparing the new product to the approved PDRN drug.

Another clinical situation characterized by a poor skin repair process and impaired angiogenesis is thermal injury. PDRN effects were investigated in mice with a deep-dermal second degree burn injury (Bitto et al., 2008a), the treatment enhanced burn wound re-epithelialization and decreased time to final wound closure. PDRN also showed a marked systemic effect: in fact, it reduced the serum levels of the pleiotropic cytokine Tumor Necrosis Factor (TNF-α) and augmented wound VEGF expression and nitric oxide production. The wound healing properties of PDRN might be the consequence of the stimulation of the altered cell-cycle machinery that is deeply impaired in several conditions: in a diabetes setting the drug stimulated the proliferation of the granulation tissue by activating cyclins driven cell-cycle progression and turning off the cell-cycle negative regulators p15 and p27 (Altavilla et al., 2011).

The ability of PDRN to promote therapeutic angiogenesis was also studied in an experimental model of peripheral artery occlusive disease induced by the excision of the femoral artery. PDRN boosted a robust blood flow restoration together with a marked increase in VEGF expression, while DMPX abrogated the beneficial effects of the drug (Bitto et al., 2008b).

Skin flap technique is commonly used in plastic surgery and esthetic medicine for a faster wound coverage, to reduce the risk of infection and restore organ function. In an experimental ischemic skin flap model PDRN increased blood flow, evaluated by laser Doppler, and again boosted a strong VEGF-driven angiogenesis (Polito et al., 2012). This result has been further confirmed in a recent paper (Chung et al., 2013), suggesting a role for this drug in improving skin flap survival. Overall, all these experimental pre-clinical observations anticipate a marked therapeutic efficacy of PDRN in a clinical setting of impaired wound healing, angiogenesis and disturbed skin repair processes.

#### PDRN anti-inflammatory activity

The adenosine A_2A_ receptor activation results in a robust anti-inflammatory effect, and it represents an interesting target for the molecular design of anti-inflammatory agents. With this scientific background, PDRN was evaluated in collagen-induced arthritis (Bitto et al., 2011). In this experimental paradigm, PDRN significantly improved the clinical signs of arthritis, reduced the histological damage and blunted the cartilage content and blood levels of several inflammatory cytokine, while increased the expression of the anti-inflammatory cytokine Interleukin-10 (IL-10). All these curative effects were abolished by the concomitant administration of DMPX, further pointing out that the adenosine A2_A_ receptor is the specific target of PDRN. The immunological and pathological processes occurring in rheumatoid arthritis and another condition named periodontitis are nearly identical. Both conditions are characterized by chronic inflammation in a soft-tissue site adjacent to bone, and periodontitis is one of the most important cause of teeth loss in adults. Experimentally periodontitis can be induced in rodents by ligation of the lower left first molar cervix. In a rat model, PRDN was tested in a gel solution applied for 7 days alone or in combination with an adenosine A2_A_ receptor antagonist. The drug reduced the histological damage, decreased the tissue levels of several inflammatory cytokines and blunted apoptotic protein expression (Bitto et al., 2013). All these effects were abrogated by the A2_A_ receptor antagonist. The treatment also markedly protected the alveolar bone quality, thus suggesting that PDRN may also promote bone regeneration, as recently confirmed by other studies (Kim et al., 2016a,b). Chronic inflammation is also deeply involved in the etiology and development of other conditions as inflammatory bowel disease and it is known that the activation of adenosine A_2A_ mitigates the inflammatory cascade in colonic epithelial cells. In agreement with this evidence, PDRN was tested in two experimental models of colitis, the drug was given by intraperitoneal injection and it was able to ameliorate tissue repair and to reduce symptomology (Pallio et al., 2016).

#### Anti-ischemic effects of PDRN

Adenosine contributes to the mechanisms underlying ischaemia/reperfusion injury and the A_2A_ receptor has been indicated as a therapeutic strategy to modulate the ischemic insult. Testicular twisting and varicocele are peculiar ischemic conditions that create a hypoxic state responsible for testicular damage and long-term complication consisting in disturbed Leydig cell activity and altered spermatogenesis. PDRN has been tested in the experimental models of testicular twisting and varicocele (Minutoli et al., 2011, 2012, 2015; Arena et al., 2012). These studies suggested that PDRN protects against testicular histological damage and markedly improves spermatogenesis, by increasing VEGF expression and angiogenesis, reducing inflammatory cascade and re-balancing the apoptotic machinery. The protective effect might be also ascribed to PDRN ability to limit ischemia reperfusion injury, as observed in the kidney (Jeong et al., 2016).

In summary, the weight of available evidence from *in vivo* studies indicates that PDRN possesses several activities: tissue repairing, anti-ischemic, and anti-inflammatory.

### Pharmacological properties of PDRN: clinical studies

#### Tissue repairing, wound healing and regenerative effects

In a first pilot study PDRN was tested in the healing of autologous skin graft donor sites. The patients were randomized into two groups: the control group received dressings with gauzes embedded in chloramine solution; the other group received the same treatment plus PDRN (5.625 mg diluted in 3 ml embedded in the gauzes) that improved re-epithelialization and, in addition, the time to complete wound healing (Valdatta et al., 2004). In a further experiment PDRN eye drops (0.75/ml) were evaluated on corneal epithelial tissue repair in patients after photorefractive keratectomy. The DNA-derived drug markedly stimulated corneal epithelium regeneration, while no significant adverse event was observed (Lazzarotto et al., 2004).

Diabetic foot ulcers are a leading cause of hospitalization and readily become chronic with poor healing properties (Falanga, 2005; Blakytny and Jude, 2006). Therefore, the opportunity to have a treatment proven to be effective for affected subjects is a breakthrough in this field. Diabetic patient with Wagner grade 1 or 2 ulcers were randomly assigned to receive placebo (*n* = 106) or PDRN (*n* = 110) for 8 weeks. The drug was injected daily by intramuscular route (5.625 mg in a 3 ml, vial) for 5 day/week and by perilesional route (5.625 mg, in a 3 ml vial) 2 day/week for 8 weeks. The treated group nearly doubled the rate of complete healing of difficult-to-heal diabetic foot ulcers compared to placebo (Table 2) as early as 8 weeks after start of treatment (Squadrito et al., 2014). This study is one of the largest trial ever carried out in diabetic patients with poor diabetic skin repair that points out a dramatic efficacy of PDRN in improving hard-to-heal chronic diabetic foot ulcers.

**Table 2:** **Outcome achieved, according to treatment group**.

	***Placebo***	***PDRN***	
Wound closure *n* (%)	20 (18.9)	41 (37.3)	*P* = 0.003
			
% epithelized area median (range)	49.3 (−160–100)	82.2 (−54–100)	*P* < 0.001
			
Time to complete wound closure (days) *n* (median; range)	48 (28–56)	53 (14–56)	*P* = 0.80

*^∧^Reported at the end of the follow-up; in 2 cases within PDRN group and in 20 cases within placebo group, ulcer area increased at the end of the follow-up*.

*Reproduced with permission from Squadrito et al. (2014)*.

The healing efficacy of the drug was also confirmed in a smaller group of patients suffering for pressure ulcers (Kim et al., 2016a). In a further study, a topical application of a gel containing PDRN and hyaluronic acid was compared with a gel containing only hyaluronic acid in patients suffering from venous ulcers of the lower limbs. The endpoint was full skin repair 45 days after the start of treatment. Complete wound healing was obtained in 67% of patients treated with PDRN and hyaluronic acid, while only 22% of patients receiving only hyaluronic acid achieved the therapy target (De Caridi et al., 2016).

Degenerative joint disease, or osteoarthritis, was successfully treated in animals with PDRN and in humans there is still a lack of an effective pharmacological therapy aimed at reducing the need of prosthesis and leading to a stabilization of the disease. The topical application of a regenerative gel containing PDRN significantly improved pain and joint mobility with a clear amelioration of the clinical sign and the radiological images (Di Nicola and Pierpaoli, 2013).

#### Other clinical evidences

Lichen sclerosus is an autoimmune inflammatory skin disease that causes a sclerosis process in the male genital tract. The available treatment for these pathological conditions consists in topical local steroids. PDRN subdermal injections (5.625 mg, in a 3 ml vial), in addition to the standard topical treatment, resulted in a marked reduction of most of the clinical signs (Laino et al., 2013), thus pointing out that intradermal administration of PDRN, together with the standard steroids treatment, may represent a promising and innovative option for this pathological conditions. This has been recently confirmed in a more recent clinical trial (Zucchi et al., 2016).

Finally, PDRN has been proven effective in the treatment (intradermal administration) of chronic plantar fasciitis (Kim and Chung, 2015) and in the therapeutic management (topical application) of female pattern hair loss (Lee et al., 2015).

### Safety

Acute and chronic toxicity studies in mice and rats were undertaken to evaluate the effects of repeated systemic administration of PDRN. PDRN (8 mg/kg) showed no toxic effect in brain, liver, lungs skeletal muscle and heart and did not cause mortality (Galeano et al., 2008). In the trial investigating the effects of PDRN on the healing of chronic diabetic foot ulcers for up to 56 days, the safety and tolerability were excellent (Squadrito et al., 2014).

Finally, post-marketing surveillance study carried out throughout 5 years and involving the selling of more than 300,000 PDRN dispensed prescriptions confirmed the excellent safety profile of the drug.

## Discussion and concluding remarks

PDRN pharmacological properties design a global picture of a drug for the management of poor wound healing caused by different pathological conditions. The lack of effects on the immune system is one of the most important determinants of the good safety profile of the drug. In this context, PDRN represents a new advancement in the pharmacotherapy. In fact adenosine and dipyridamole are non-selective activators of adenosine receptors and they may cause unwanted side effects. Indeed, regadenoson is the only other one A_2A_ receptor agonist available in the market but has been approved by the FDA as a pharmacological stress agent in myocardial perfusion imaging, a well-established noninvasive modality for the diagnosis and prognosis of coronary artery disease (Al Jaroudi and Iskandrian, 2009; Bengalorkar et al., 2012; Reyes, 2016). It displays fewer side effects that adenosine or dipyridamole, but its potential use in other pathological conditions has not yet explored.

Besides engaging the A_2A_ receptor, PDRN offers nucleosides and nucleotides for the so called “salvage pathway.” The tissue repairing and healing effects are unique features of PDRN that are not shared with other DNA derived drug from different origin, molecular weight and manufacturing process. Therefore, caution must be used when extrapolating the pharmacological properties of PDRN to other DNA derived products.

## Author contributions

FS contributed to conception, drafting the article, and final approval; AB contributed substantially to revision for important intellectual content and final approval of the version to be published; NI and GPa made substantial contributions to revising the article; GPi made substantial contributions to revising the article; LM and DA contributed substantially to revision for important intellectual content and final approval of the version to be published. All the authors gave final approval of the version to be published.

## Funding

This paper was supported by Departmental funding assigned to Prof. FS.

### Conflict of interest statement

The authors declare that the research was conducted in the absence of any commercial or financial relationships that could be construed as a potential conflict of interest.
