# Corrigendum: Pharmacological activity and clinical use of PDRN

**DOI:** 10.3389/fphar.2022.1073510

**Published:** 2022-11-21

**Authors:** Francesco Squadrito, Alessandra Bitto, Natasha Irrera, Gabriele Pizzino, Giovanni Pallio, Letteria Minutoli, Domenica Altavilla

**Affiliations:** ^1^ Section of Pharmacology, Department of Clinical and Experimental Medicine, University of Messina, Messina, Italy; ^2^ Department of Biomedical and Dental Sciences and Morphofunctional Imaging, University of Messina, Messina, Italy

**Keywords:** adenosine A2 receptors, DNA, oligonucleotides, PDRN, wound healing

In the published article, the **Conflict of Interest** statement was incorrectly filed. Disclaimers on patents were omitted from the article. The correct Conflict of Interest statement appears below.

“Author FS has received research support from Mastelli for work on polydeoxyribonucleotide. Authors FS, AB, and DA are co-inventors on a patent describing therapeutic polydeoxyribonucleotide activity in chronic intestinal disease. Author LM is a co-inventor on a patent describing therapeutic polydeoxyribonucleotide activity in testicular injury by torsion.

The remaining authors declare that the research was conducted in the absence of any commercial or financial relationships that could be construed as a potential conflict of interest.”

The authors apologize for this error and state that this does not change the scientific conclusions of the article in any way. The original article has been updated.

